# Hook Worm Eradication

**Published:** 1910-06

**Authors:** L. J. Sharp

**Affiliations:** Commerce, Ga.


### HOOK WORM ERADICATION.

BY L. J. SHARP, M. D., COMMERCE, GA.

A small worm whose favorite abode is in the upper alimentary canal, was named hook worm by its first discoverer 330 years ago, another independent discoverer named it Uncinaria, and another, Anchilostoma. The most common type was named by Dr. Stiles, Necator Americanus (American murderer).

I have had very little experience with hook worm disease, but think that I see a great many cases, although my practice is in a section of the state where this disease is least prevalent. I have inquired of neighboring physicians of large practice about cases, and they say they have had very few, some none. I have treated about a dozen cases. One was one of my farm hands, who lost two months' work and had to be forced by his parents to take treatment. Another was sent to me by a landlord to be treated for ulcer of the leg. She belonged to a large family, and all but one appear to have it. Only one other of this family has taken treatment, and she, as well as all other cases I have treated, were required to by their parents, because they became too weak to work. None have applied for treatment or sent speciment for free examination of their own accord.

They are a very indifferent set, and seem to be affected with a similar mental state of some belonging to our profession, as well as others who seem to take pride in publishing in the lay press their want of faith in the lazy worm ideas. These are a great stumbling block in the way of hook worm eradication. Cases of Uncinariasis frequently give quotations from these when the subject of hook worm is mentioned. When I see a suspect and opportunity presents, I usually tell them their palor can probably be easily cured by their family physician at a small expense. None of these have shown any improvement except those I treated, hence I judge the others have taken no treatment. Evidently some relation exists between this disease and this state of indifference, hence I think the public should take special interest in them. Many of you have been too busy to keep up with the literature on this subject, and I will abbreviate some of it bearing upon this subject of eradication.

The first question that presents itself is: Does this worm do enough harm to justify the effort necessary for its eradication?

Doubtless this disease has existed since the origin of man. It was described accurately in Egyptian literature about 3500 years ago. Only in the latter part of the last eight years has any special attention been paid to it. Dr. Stiles says he incidentally came across a family where 15 had died and the remaining 5 had hook worm. The parents said the 15 died with the same disease that the 5 had. He visited 130 Southern cotton mills and diagnosed Uncinariasis in one out of 8 operatives on inspection; the microscope would reveal eggs in from 50 to 70 per cent. Dr. Newton Evans examined 147 soldiers apparently healthy and found 64 had hook worm. Chamberlain found 60 in 100, in a State Normal School at Austin, Texas, 31 were found in 100; of the medical students at Tulane, New Orleans, 38 were found in 100. Dr. Schnauss, of Cecil, Ga., says he had 849 cases in 3 years. Dr. A. G. Fort, of Lumpkin, Ga., says he has treated 600. Many report large numbers of fatal cases due to hook worm alone, and many more succomb when complicated by other diseases.

Doctors Stiles and Harris say there are more cases of Uncinariasis than any other infectious disease in the South. We also see that it produces mental and physical weakness, prevents proper development, injures those most that are the most dependent and are the hope of every country—the children. Would you say "a little more sleep, a little more slumber," when there is such a subtile and destructive foe at your neighbor's door, if not our own? It does not attack with a great display of arms or noisy implements of warfare, but casts off great numbers of invisible eggs daily in human discharge. In rural sections, are strewn over hills and valleys, by the highways and hedges, scattered by everything that comes in contact with them; and the little worm that soon escapes can remain for a long time awaiting in ambush and will penetrate the skin of the first human with which it comes in contact, plows through the tissues of the body, lodges in its favorite abode in the small intestines where it saps the life fluids from and poisons its subject.

Their eggs must be discharged from the body to be hatched and developed. This gives us a fine opportunity for their eradication. They prefer a warm climate, sandy, loose, shady and moist soil.

We have ample reason for making war against the hook worm alone, but since the typhoid germ and a few others are cast off and spread in similar way, we have more than sufficient reason, and we should destroy these germs immediately after being discharged.

Washington health report of March 18th says recent examinations of varying numbers of rural homes from 110 to 135, in North Carolina, 62 per cent. had no privies; in South Carolina, 65 per cent.; in Georgia, 70 per cent.; in Florida, 30 per cent., 2 families of whites to 1 of blacks had privies. The population of whites to negroes in the South is about 5 to 4. Negroes that have had examinations show a larger proportion infected with hook worm. Animals have hook worm, but a different type, hence all human discharges should be properly looked after.

Teach the public first, not only the cause of Uncinariasis but the cause and means of prevention of all contagious and infectious diseases. Have the government issue a brief book on sanitation, and require all teachers to pass a thorough examination on this book; teach it exclusively to all the pupils during the first week of each term, and each student be required to pass a satisfactory examination at the end of the first week in each term or get no benefit of the public fund. Invite and admit the public free first week, and have night lectures for the general public. During or before this first week have ample and proper privy accommodations; have wells and springs covered insect proof; have all undergrowth or other obstructions to free access of air, sunlight and public view removed from the school grounds a distance of one-fourth of a mile if practicable.

Require certificate from a qualified medical examiner that the school applicant has not been recently exposed to contagious disease, and that there are no typhoid or tubercle baccilli or hook worm eggs thrown off. All this should be free of expense to the applicant. Mailing cases for sending specimens should be furnished free to any one that asks for them and continue free examinations by boards of health. Make it unlawful for any one to pollute school buildings, yards, play grounds or water supply with any human discharge and that all such shall be covered. Dogs and cats do that much. Where soil is infected with hook worm, recommend that shoes be worn and that the skin of every one be kept free from dirt, especially children. Require the disinfection of sewerage and the proper construction of privy boxes.

Large sums of money are spent annually by incorporated towns to no benefit on account of improperly constructed privies. The book on sanitation should illustrate and instruct how to make a privy box. I have investigated and decided that the best thing to my mind, all things considered, for a rural privy is a seat, with opening, fastened to the top of a galvanized box or tub with a loose pin X-hinge, and a close-fitting lid hinged over this so neither can be left open or allow entrance of insects. If far enough from any one's water supply it could be placed over a deep dry well in which the discharges will soon liquify and disappear. One or more wells could be some distance from the closet to escape polluting water and the box be carried and emptied into it and covered with ashes or dirt each time. The lid on the box can be removed and replaced by means of the loose pin in the hinge.

The lay press should be supplied with such matter as should be published, and this should be the special duty of those that are authorized to do such work, to whom all should submit everything before publication. The names of suspects should be sent to boards of health.

Hook worms have been proven to live 11 years, and many live many years longer, so we should destroy them, which can be easily done in the human with Thymol. Hook worm should be added to the list of diseases for which treatment is furnished free to the poor. Thanks and honor to our Legislature for their good work on this line.

Treatment need be taken only one day each week until no eggs are found under the microscope, or worms found when feces are washed through cheese cloth. Remmeber, Thymol is soluble in oils and alcoholics, if dissolved, can poison the patient instead of the hook worm. Remember, oils are in butter, sweet milk, soups, meats, etc.; alcohol, in drugs, drinks, etc. Nothing should be taken for six hours before and six hours after Thymol except by a physician's instruction.

Give salts or calomel Saturday night omitting supper. Next morning at 6, 7 and 8 o'clock give in capsule ½ gr. Thymol each dose for every year of age, or more to the vigorous, salts or calomel with the last dose, light food at 9 or later, and if no move by 12, give enema.

No one has more influence in a medical way over a family than their physician, so we ask you to go out in this missionary field, teaching cleanliness, which is next to gasoline. You may fail in part, so did Moses and the prophets.

